# Usefulness of health checkup for screening metabolic dysfunction-associated fatty liver disease and alcohol-related liver disease in Japanese male young adults

**DOI:** 10.1038/s41598-023-34942-x

**Published:** 2023-05-18

**Authors:** Satoko Tajirika, Takao Miwa, Cathelencia Francisque, Tatsunori Hanai, Nanako Imamura, Miho Adachi, Ryo Horita, Lynette J. Menezes, Masahito Shimizu, Mayumi Yamamoto

**Affiliations:** 1 Health Administration Center, Gifu University, 1-1 Yanagido, Gifu, 501-1193 Japan; 2 Department of Gastroenterology/Internal Medicine, Graduate School of Medicine, Gifu University, 1-1 Yanagido, Gifu, 501-1194 Japan; 3 Morsani College of Medicine, University of South Florida, 12901 Bruce B. Downs Blvd., Tampa, FL 33612 USA; 4 Department of Diabetes and Metabolism, Gifu University Hospital, Gifu, 1-1 Yanagido, 501-1194 Japan; 5 United Graduate School of Drug Discovery and Medical Information Sciences, Gifu University, Gifu, 1-1 Yanagido, 501-1194 Japan

**Keywords:** Gastroenterology, Hepatology, Disease prevention, Public health

## Abstract

We aimed to assess metabolic dysfunction-associated fatty liver disease (MAFLD) and alcohol-related liver disease (ALD) prevalence in young male adults and the role of health checkups in disease screening. We recruited 313 male graduate students at Gifu University in April 2022. With hepatic steatosis diagnosed by ultrasonography, MAFLD and nonalcoholic fatty liver disease (NAFLD) were diagnosed based on health checkup data, and ALD was diagnosed with alcohol consumption > 30 g/day. The ability of each variable to identify MAFLD, NAFLD, and ALD was assessed using logistic regression and receiver-operating characteristic curve analyses. Participants’ mean age was 23 (± 4) years, and MAFLD, NAFLD, and ALD prevalence was 11%, 17%, and 1%, respectively. Among Japanese male young adults, alanine aminotransferase (ALT) (odds ratio [OR] 1.04; 95% confidence interval [CI] 1.01–1.07; *P* = 0.008) and body mass index (BMI) (OR 2.02; 95% CI 1.58–2.58; *P* < 0.001) were independently associated with MAFLD. Furthermore, only the alcohol use disorders identification test (AUDIT) was able to identify ALD (OR 1.49; 95% CI, 1.28–1.74; *P* = 0.001). Our study revealed that health checkups, including measurement of ALT, BMI, and AUDIT, are important for screening MAFLD and ALD in younger generations.

## Introduction

Chronic liver disease (CLD), characterized by chronic liver inflammation, affects 1.5 billion people globally, accounting for nearly 2 million deaths due to liver cancer and cirrhosis^1^. Although CLD remains asymptomatic for a long period, when it eventually progresses to cirrhosis, patients experience painful clinical conditions, such as malnutrition, sarcopenia, varices, ascites, and hepatic encephalopathy, which result in impaired quality of life, frequent hospital admission, and poor prognosis^2,3^. The etiology of CLD is dramatically shifting from hepatitis B/hepatitis C viral infections to alcohol-related liver disease (ALD) or nonalcoholic fatty liver disease (NAFLD) due to the implementation of hepatitis B vaccination, advances in hepatitis C treatment, increasing obesity and metabolic syndrome incidence, and rising alcohol misuse^1^. The Global Burden of Disease data analysis demonstrated that the cause of CLD among 15–29-years-olds has also shifted worldwide from hepatitis B infections to NAFLD^4^. Therefore, early detection and intervention of NAFLD and ALD is an unmet clinical need, especially in young adults, for reducing CLD burden.

With the accumulated evidence of NAFLD, a panel of international experts from 22 countries proposed “metabolic dysfunction-associated fatty liver disease” (MAFLD) as a new term to identify patients with fatty liver disease at risk for severe outcomes^5,6^. Therefore, MAFLD is diagnosed with hepatic steatosis and the presence of at least one risk factor, such as obesity, type 2 diabetes mellitus, and metabolic dysregulation^5,6^. Recent evidence showed that the MAFLD criteria identified patients with more advanced liver fibrosis and worse prognosis than the NAFLD criteria only^7,8^. Since the diagnosis of MAFLD does not rely on alcohol consumption, recent studies emphasize that alcohol consumption is an independent factor for stratifying the risk of liver fibrosis and mortality in CLD^7,9^. Although adverse effects of MAFLD on CLD have been demonstrated^7,8^, very few attempts have been made on screening and assessing the prevalence of MAFLD among young adults. Additionally, although MAFLD was associated with the worsening symptoms of CLD, guidelines or best practices for the relatively new criteria have not been established.

In Japan, a mandatory annual health checkup for all populations has been established and provided even for young adults, based on the Health and Safety Act and the Occupational Health Act. The primary aim of this study was to determine the prevalence of MAFLD and ALD in young Japanese male adults. The secondary aim was to establish robust screening methods for MAFLD and ALD during the annual health checkups.

## Methods

### Study design and samples

This cross-sectional study included male graduate students who had entered Gifu University (Gifu, Japan) and underwent an annual health checkup for freshmen in a graduate program in April 2022. The following inclusion criteria were used: an incoming student at Gifu University Graduate Program of 2022, aged 18 years or older, self-identification as Japanese, and completion of all examination items including blood sampling and health questionnaire that included present medical treatment. All male students attended the health checkup underwent abdominal ultrasonography (CX50; Koninklijke Philips N.V., Amsterdam, Netherlands) administered by a hepatologist completely blinded to the results of this study, along with a questionnaire on alcohol consumption. Biochemical assessments included aspartate aminotransferase (AST), alanine aminotransferase (ALT), triglycerides (TG), high-density lipoprotein cholesterol (HDL-C), low-density lipoprotein cholesterol, hemoglobin A1c (HbA1c), and fasting or random glucose levels.

**Figure 1:**
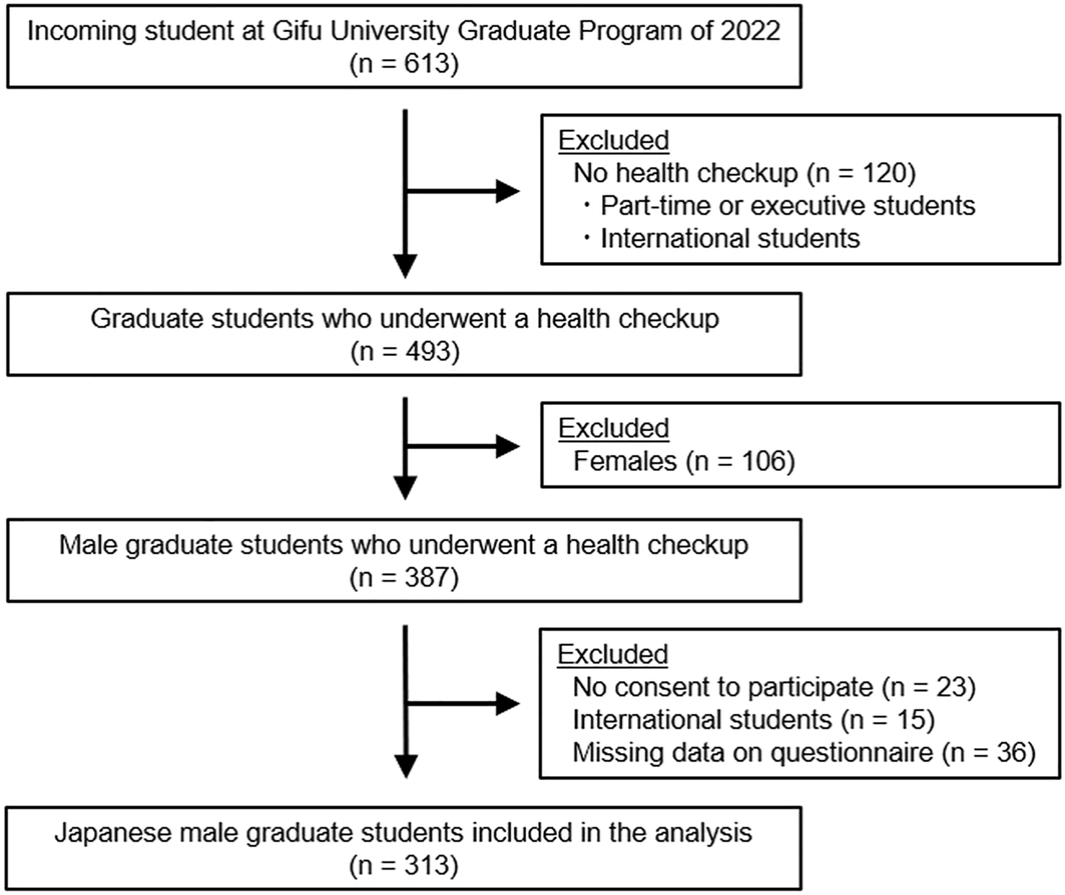
A flow diagram of the study.

Of the 613 incoming students at Gifu University Graduate Program of 2022, 120 had no annual health checkup because these students were part-time or executive students who have a health checkup at their workplace or international student who could not attend the scheduled health checkup. In addition, 106 females were initially excluded from the study and 60 students were excluded based on the inclusion and exclusion criteria. Finally, 313 Japanese male graduate students were included in the analysis (Fig. 1).

### Diagnosis of NAFLD and MAFLD

NAFLD was diagnosed based on the presence of liver steatosis and excluding ALD, viral liver disease, and drug-induced liver injury, according to the guidelines for NAFLD^10,11^.

The diagnosis of MAFLD was based on the diagnostic criteria proposed by an international expert panel and was diagnosed in participants with hepatic steatosis by ultrasonography and one of the following criteria: overweight or obesity, type 2 diabetes mellitus, and metabolic dysregulation^5^. Overweight or obesity for MAFLD diagnosis was defined as body mass index (BMI) ≥ 23 kg/m^2^ according to the Asian cutoff value. Type 2 diabetes mellitus was defined as HbA1c ≥ 6.5% or use of a specific anti-diabetic treatment. Metabolic dysregulation was defined as the presence of at least two of the following criteria: waist circumference ≥ 90 cm, blood pressure ≥ 130/85 mmHg, TG level ≥ 150 mg/dl, HDL-C level < 40 mg/dl, prediabetes with fasting blood glucose from 100 to 125 mg/dL, random blood glucose 140–199 mg/dL, HbA1c from 5.7 to 6.4%, or any specific treatment related to these conditions^5^.

### Diagnosis of ALD and assessment of excessive alcohol intake

The diagnosis of ALD was based on the standard guidelines^12,13^. Participants with liver steatosis and regular alcohol consumption of > 30 g/day were diagnosed with ALD according to the reference data for males^12,13^.

Alcohol consumption was assessed using a health questionnaire during the health checkups. The participants were asked the following question: “How many days per week do you consume alcohol on average?” (Choices: 0 days, 1–2 days, 3–4 days, 5–6 days, everyday) “How many cups of sake do you consume at one opportunity of drinking?” (Choices: less than 1, 1, 2, 3, more than 3). A cup (180 mL) of sake corresponds to one-quarter bottle of wine (180 mL of 14% alcohol). Daily alcohol consumption was calculated based on the frequency of alcohol consumption and amount of alcohol consumption during a single sitting, and participants with an alcohol intake of ≥ 20 g/day were assessed as having excessive alcohol intake based on the Japanese reference data^14^.

The alcohol use disorders identification test (AUDIT) was administered to all participants as a self-reported questionnaire according to the guidelines of ALD^12,13^. The AUDIT consists of 10 questions on consumption (Q1–Q3), dependence symptoms (Q4–Q6), and alcohol-associated problems (Q7–Q10), and each question is scored from 0 to 4, as previously reported^15^. AUDIT-consumption (AUDIT-C) was assessed by the first three questions of AUDIT (Q1–Q3) as a shortened version^12,13^.

### Statistical analysis

Continuous variables are presented as mean and standard deviation. Categorical variables are presented as numbers and percentages (%). The two groups were compared using the chi-square test or Mann–Whitney *U* test. The Kruskal–Wallis test was used for multiple pairwise comparisons. Predictors of NAFLD, MAFLD, and excessive alcohol intake were assessed using logistic regression analysis and expressed as odds ratios (ORs) with 95% confidence intervals (CIs). Predictors with *P* values < 0.05 in the individual analysis were included in the multivariable analysis, considering the importance of variables and confounders. The discriminative ability of each variable was assessed using receiver operating characteristic (ROC) curve analysis and was presented as the area under the curve (AUC) with 95% CI. The optimal cutoff value was estimated using the Youden index value, and discriminative ability was shown by sensitivity, specificity, positive predictive value (PPV), and negative predictive value (NPV). The significance threshold was set at *P* < 0.05. Statistical analyses were performed using JMP version 16.2.0 software (SAS Institute Inc., Cary, NC, USA) and R version 4.1.2 software (The R Foundation for Statistical Computing, Vienna, Austria).

### Ethical considerations

All study procedures complied with the ethical requirements of the national and institutional committees that oversee human studies, and the 1964 Declaration of Helsinki and its later revisions. The study design was reviewed and approved by the Ethics Review Committee of the Graduate School of Medicine, Gifu University, Japan (approval no. 2021-B167). The study design and objective were fully explained, and data were collected after obtaining written informed consent from all participants.

## Results

### Prevalence of NAFLD and MAFLD

**Figure 2:**
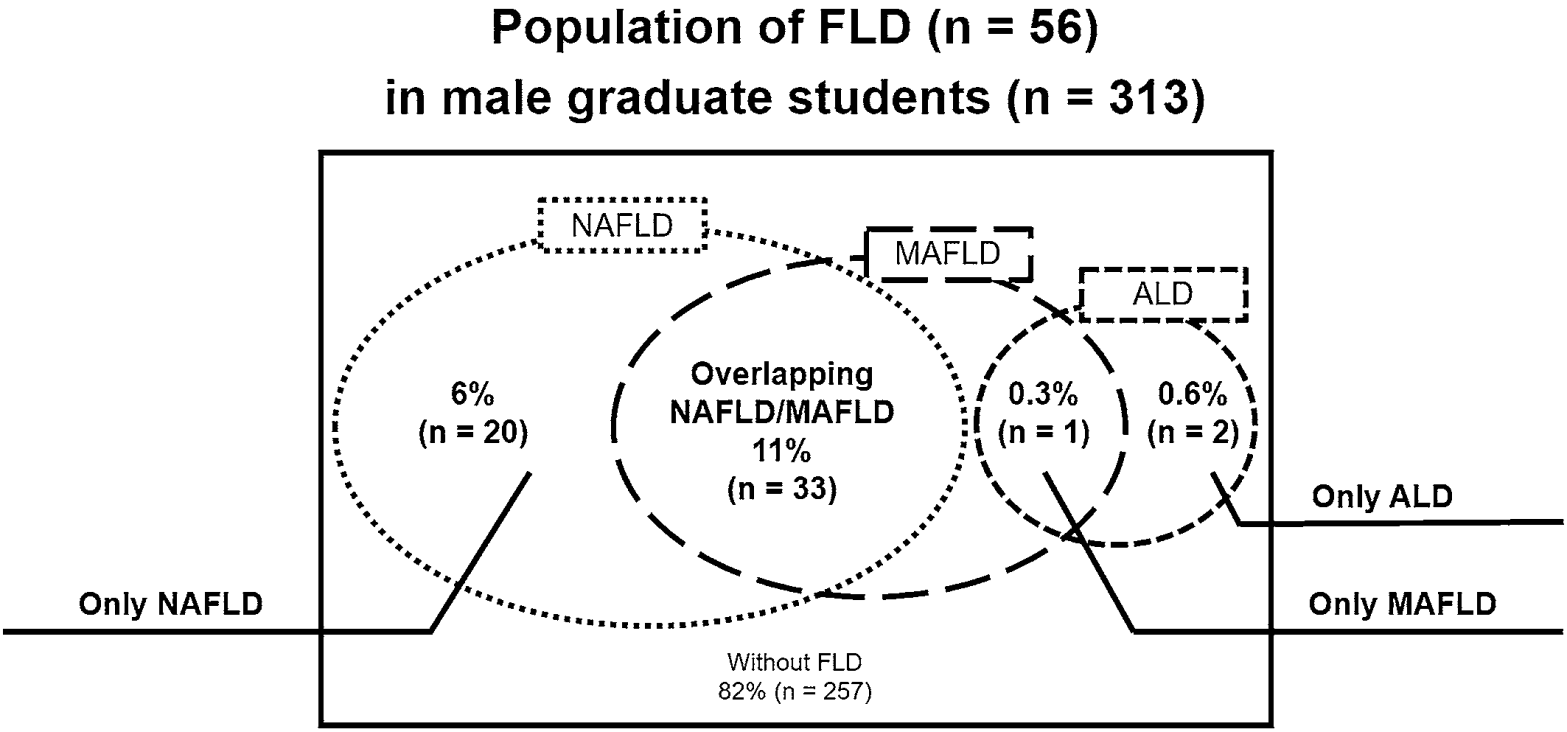
The population of FLD (n = 56) among male graduate students (n = 313). Abbreviations: *ALD* alcohol-related liver disease; *FLD* fatty liver disease; *MAFLD* metabolic dysfunction-associated fatty liver disease; *NAFLD* nonalcoholic fatty liver disease.

The characteristics of the participants and comparisons between those with and without NAFLD or MAFLD are presented in Table 1. The mean age and BMI of the 313 male graduate students were 23 (± 4) years and 21.2 (± 2.9) kg/m^2^, respectively. Of the participants, 56 (18%) had hepatic steatosis and NAFLD, MAFLD, and ALD were diagnosed in 53 (17%), 34 (11%), and 3 (1%) of the participants, respectively (Tables 1 and 2). Of these participants, overlapping NAFLD and MAFLD were observed in 33 (11%), whereas 20 (6%) had only NAFLD, and one (0.3%) had only MAFLD. Furthermore, one participant (0.3%) had overlapping MAFLD and ALD, whereas two (0.6%) had only ALD (Fig. 2).

**Table 1:** Characteristics of participants with MAFLD and NAFLD.

Characteristic	All participants (n = 313)	MAFLD (n = 34)	No MAFLD (n = 279)	*P* value	NAFLD (n = 53)	No NAFLD (n = 260)	*P* value
Demographics							
Age (years)	23 (± 4)	25 (± 7)	23 (± 3)	0.011	24 (± 6)	23 (± 3)	0.149
Current/former smoking, n (%)	24 (8)	1 (3)	23 (8)	0.273	4 (8)	20 (8)	0.971
Excessive alcohol intake, n (%)	11 (4)	1 (3)	10 (4)	0.848	1 (2)	10 (4)	0.480
Exercise habits, n (%)	123 (39)	13 (38)	110 (39)	0.893	20 (38)	103 (40)	0.799
Physical examination							
Waist circumference (cm)	78 (± 8)	90 (± 12)	76 (± 6)	< 0.001	85 (± 12)	76 (± 6)	< 0.001
Body mass index (kg/m^2^)	21.2 (± 2.9)	26.3 (± 3.3)	20.6 (± 2.2)	< 0.001	24.3 (± 3.8)	20.6 (± 2.3)	< 0.001
Comorbidity							
Diabetes, n (%)	0 (0)	0 (0)	0 (0)	NA	0 (0)	0 (0)	NA
Overweight or obesity, n (%)	67 (21)	30 (88)	37 (13)	< 0.001	29 (55)	38 (15)	< 0.001
Metabolic risk abnormality							
High waist circumference, n (%)	28 (9)	16 (47)	12 (4)	< 0.001	15 (28)	13 (5)	< 0.001
High blood pressure, n (%)	76 (24)	25 (74)	51 (18)	< 0.001	28 (53)	48 (18)	< 0.001
High TG, n (%)	45 (14)	18 (53)	27 (10)	< 0.001	21 (40)	24 (9)	< 0.001
Low HDL-C, n (%)	13 (4)	6 (18)	7 (3)	< 0.001	6 (11)	7 (3)	0.004
Prediabetes, n (%)	6 (2)	2 (6)	4 (1)	0.074	2 (4)	4 (2)	0.279
Laboratory test							
AST (U/L)	21 (± 13)	29 (± 20)	20 (± 12)	< 0.001	26 (± 17)	20 (± 12)	< 0.001
ALT (U/L)	26 (± 21)	56 (± 47)	22 (± 11)	< 0.001	46 (± 41)	22 (± 11)	< 0.001
TG (mg/dL)	99 (± 70)	189 (± 142)	88 (± 45)	< 0.001	157 (± 126)	88 (± 44)	< 0.001
HDL-C (mg/dL)	58 (± 13)	50 (± 11)	59 (± 13)	< 0.001	54 (± 12)	59 (± 13)	0.009
LDL-C (mg/dL)	97 (± 26)	103 (± 24)	96 (± 26)	0.050	96 (± 25)	97 (± 26)	0.871
HbA1c (%)	5.2 (± 0.2)	5.3 (± 0.2)	5.2 (± 0.2)	< 0.001	5.3 (± 0.2)	5.2 (± 0.2)	< 0.001
AUDIT	3.8 (± 4.3)	3.9 (± 4.4)	3.8 (± 4.3)	0.956	3.5 (± 3.6)	3.9 (± 4.5)	0.794
Q1–Q3 (AUDIT-C)	2.9 (± 2.6)	2.9 (± 2.6)	2.9 (± 2.6)	0.963	2.8 (± 2.3)	3.0 (± 2.7)	0.898
Q4–Q6	0.4 (± 1.0)	0.4 (± 1.1)	0.4 (± 0.9)	0.407	0.3 (± 0.9)	0.4 (± 1.0)	0.143
Q7–Q10	0.5 (± 1.5)	0.6 (± 1.6)	0.5 (± 1.4)	0.850	0.4 (± 1.4)	0.5 (± 1.5)	0.382

Values are presented as numbers (percentages) or means (standard deviations). Statistical differences between the two groups were analyzed using the chi-square test or Mann–Whitney *U* test.

*ALT* alanine aminotransferase, *AST* aspartate aminotransferase, *AUDIT* alcohol use disorder identification test, *AUDIT-C* alcohol use disorder identification test-consumption, *HbA1c* hemoglobin A1c, *HDL-C* high-density lipoprotein cholesterol, *LDL-C* low-density lipoprotein cholesterol, *MAFLD* metabolic dysfunction-associated fatty liver disease, *NAFLD* non-alcoholic fatty liver disease, *TG* triglycerides.

**Table 2:** Characteristics of participants with ALD.

Characteristic	ALD (n = 3)	No ALD (n = 310)	*P* value
Demographics			
Age (years)	22 (± 1)	23 (± 4)	0.922
Current/former smoking, n (%)	0 (0)	24 (8)	0.616
Excessive alcohol intake, n (%)	3 (100)	8 (3)	< 0.001
Exercise habits, n (%)	0 (0)	123 (40)	0.161
Physical examination			
Waist circumference (cm)	80 (± 9)	78 (± 8)	0.689
Body mass index (kg/m^2^)	21.9 (± 3.8)	21.2 (± 2.9)	0.780
Comorbidity			
Diabetes, n (%)	0 (0)	0 (0)	NA
Overweight or obesity, n (%)	1 (33)	66 (21)	0.613
Metabolic risk abnormality			
High waist circumference, n (%)	1 (33)	27 (9)	0.137
High blood pressure, n (%)	2 (67)	74 (24)	0.085
High TG, n (%)	1 (33)	44 (14)	0.347
Low HDL-C, n (%)	0 (0)	13 (4)	0.717
Prediabetes, n (%)	0 (0)	6 (2)	0.808
Laboratory test			
AST (U/L)	20 (± 4)	21 (± 13)	0.466
ALT (U/L)	29 (± 11)	26 (± 21)	0.214
TG (mg/dL)	110 (± 51)	99 (± 70)	0.499
HDL-C (mg/dL)	62 (± 18)	58 (± 13)	0.453
LDL-C (mg/dL)	95 (± 33)	97 (± 25)	0.771
HbA1c (%)	5.1 (± 0.2)	5.2 (± 0.2)	0.506
AUDIT	16.7 (± 4.5)	3.7 (± 4.1)	0.004
Q1–Q3 (AUDIT-C)	8.3 (± 2.9)	2.9 (± 2.6)	0.011
Q4–Q6	3.0 (± 1.0)	0.4 (± 0.9)	< 0.001
Q7–Q10	5.3 (± 1.5)	0.5 (± 1.4)	< 0.001

Values are presented as numbers (percentages) or means (standard deviations). Statistical differences between the two groups were analyzed using the chi-square test or Mann–Whitney *U* test.

*ALD* alcohol-related liver disease; *ALT* alanine amino transferase; *AST* aspartate aminotransferase; *AUDIT* alcohol use disorders identification test; *AUDIT-C* alcohol use disorders identification test-consumption; *HbA1c* hemoglobin A1c; *HDL-C* high-density lipoprotein cholesterol; *LDL-C* low-density lipoprotein cholesterol; *TG* triglycerides.

Participants with MAFLD had a significantly higher waist circumference and BMI than those without MAFLD. They also had a higher prevalence of hypertension, hypertriglyceridemia, as well as lower HDL-C levels than those without MAFLD. Biochemical data showed significantly increased values of AST, ALT, TG, and HbA1c and decreased HDL-C levels in participants with MAFLD compared to those without MAFLD. Similar results were obtained in the comparison between participants with and without NAFLD, except for HDL-C level (Table 1).

### Prevalence of ALD and excessive alcohol intake

ALD was observed in three participants (1%), who had significantly higher AUDIT (16.7 points vs. 3.7 points; *P* = 0.004) and AUDIT-C (8.3 points vs. 2.9 points; *P* = 0.011) scores than those without ALD. However, no significant difference was found in clinical variables, including biochemical analysis data, between the two groups (Table 2). Contrastingly, excessive alcohol intake (≥ 20 g/day) was observed in 11 participants (4%), and there was no significant difference in each characteristic except for AUDIT and AUDIT-C scores between the excessive and normal groups, as shown in Supplementary Table S1.

### Screening of MAFLD and NAFLD by serum ALT levels and BMI

**Figure 3:**
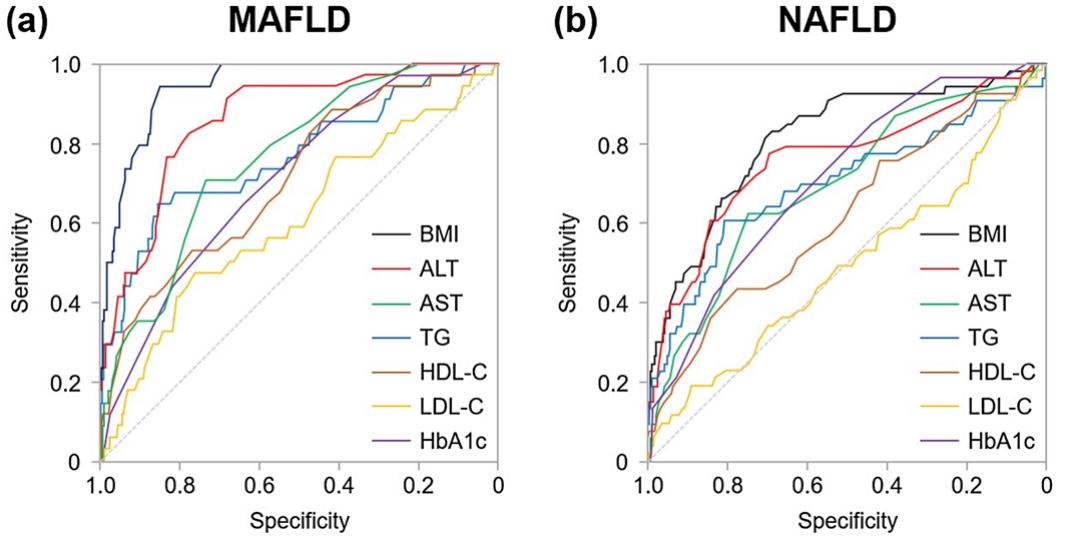
The comparison of AUC of each biochemical parameter to identify (**a**) MAFLD and (**b**) NAFLD. Abbreviations: *ALT* alanine aminotransferase; *AST* aspartate amino transferase; *AUC* under the curve; *HbA1c* hemoglobin A1c; *HDL-C* high-density lipoprotein cholesterol; *LDL-C* low-density lipoprotein cholesterol; *MAFLD* metabolic dysfunction-associated fatty liver disease; NAFLD, nonalcoholic fatty liver disease; TG, triglycerides.

**Figure 4:**
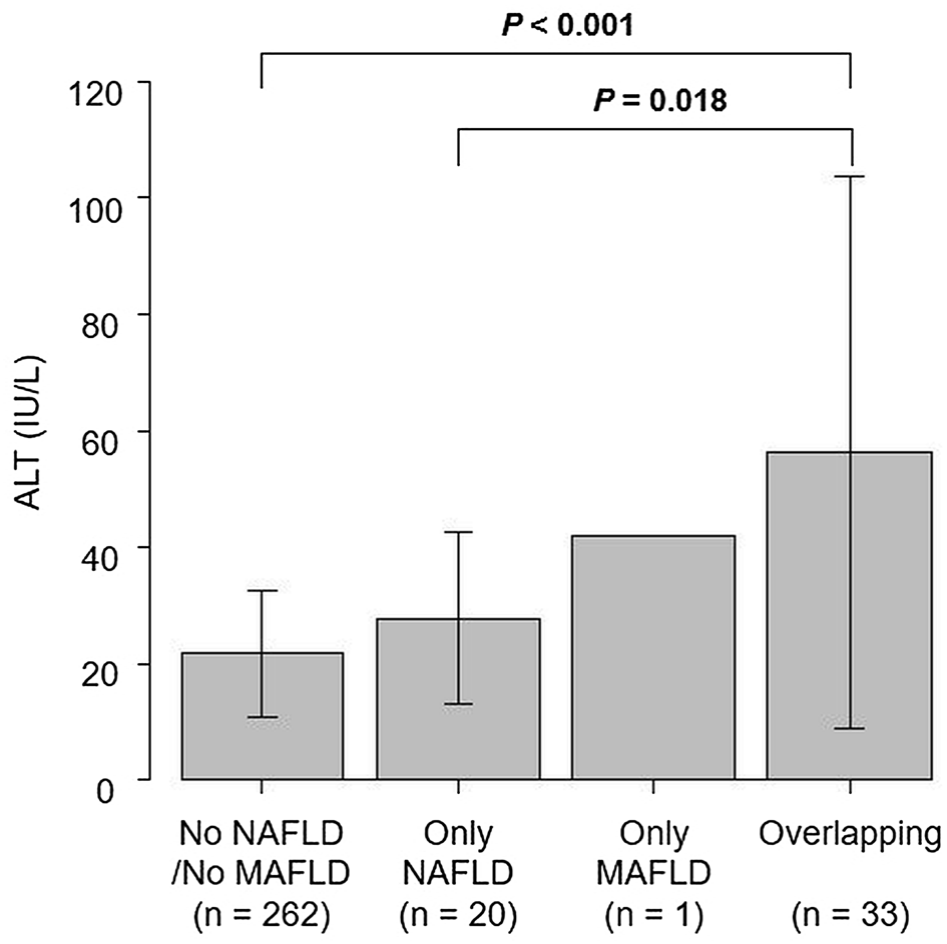
Difference of serum ALT levels between MAFLD and NAFLD. Abbreviations: *ALT* alanine aminotransferase; *MAFLD* metabolic dysfunction-associated fatty liver disease; *NAFLD* nonalcoholic fatty liver disease.

To identify a robust marker for MAFLD or NAFLD screening, multivariable analyses were performed using age, smoking, alcohol consumption, exercise, BMI, and serum ALT levels. As shown in Table 3, ALT (OR 1.04; 95% CI 1.01–1.07; *P* = 0.008) and BMI (OR 2.02; 95% CI 1.58–2.58; *P* < 0.001) were independently associated with MAFLD. The ROC analysis showed that serum ALT levels and BMI had the larger AUC than other biochemical parameters for identifying MAFLD (Fig. 3a). Similar results were obtained for the association between ALT (OR 1.04; 95% CI 1.02–1.07; *P* = 0.001), BMI (OR 1.43; 95% CI 1.24–1.64; *P* < 0.001), and NAFLD (Fig. 3b). Details of the individual analysis are presented in Supplementary Table S2. The optimal cutoff values of ALT for identifying MAFLD and NAFLD were 26 U/L and 23 U/L, respectively. With the ALT cutoff value to identify MAFLD, the sensitivity, specificity, PPV, and NPV were 0.82, 0.77, 0.31, and 0.97, respectively. Similarly, the sensitivity, specificity, PPV, and NPV of ALT to identify NAFLD were 0.94, 0.34, 0.69, and 0.77, respectively. Furthermore, participants with overlapping NAFLD and MAFLD had significantly higher serum ALT levels than those without NAFLD/MAFLD overlap and NAFLD only (Fig. 4). In addition, the discriminative ability of BMI to identify MAFLD and NAFLD were also evaluated and the optimal cutoff values were 22.9 kg/m^2^ and 21.5 kg/m^2^, respectively. With the BMI cutoff value to identify MAFLD, the sensitivity, specificity, PPV, and NPV were 0.94, 0.85, 0.43, and 0.99, respectively. Similarly, the sensitivity, specificity, PPV, and NPV of BMI to identify NAFLD were 0.81, 0.70, 0.36, and 0.95, respectively.

**Table 3:** Multivariable analysis of the factors associated with MAFLD and NAFLD among participants.

Characteristic	OR (95%CI)	*P* value
MAFLD		
Age (years)	1.05 (0.94–1.19)	0.053
Current/former smoking	0.14 (0.01–3.08)	0.213
Excessive alcohol intake	0.52 (0.03–8.67)	0.519
Exercise habits	0.66 (0.22–2.00)	0.466
Body mass index (kg/m^2^)	2.02 (1.58–2.58)	< 0.001
ALT (U/L)	1.04 (1.01–1.07)	0.008
NAFLD		
Age (years)	1.03 (0.94–1.12)	0.497
Current/former smoking	1.06 (0.29–3.84)	0.927
Excessive alcohol intake	0.39 (0.04–3.90)	0.425
Exercise habits	0.78 (0.37–1.63)	0.510
Body mass index (kg/m^2^)	1.43 (1.24–1.64)	< 0.001
ALT (U/L)	1.04 (1.02–1.07)	0.001

Analyses were performed using logistic regression models.

*ALT* alanine aminotransferase, *CI* confidence interval, *MAFLD* metabolic dysfunction-associated fatty liver disease, *NAFLD* non-alcoholic fatty liver disease, *OR* odds ratio.

### Screening of ALD and excessive alcohol intake by the AUDIT or AUDIT-C

Among all participants, although clinical and biochemical parameters had no association with ALD, the AUDIT (OR 1.49; 95% CI 1.28–1.74; *P* = 0.001) and AUDIT-C (OR, 1.85; 95% CI, 1.20–1.74; *P* = 0.005) scores had good discriminative ability to identify ALD with AUCs of 0.98 (95% CI 0.96–1.00) and 0.92 (95% CI 0.80–2.86), respectively (Supplementary Table S3). With an optimal cutoff value of 12 points for AUDIT, the sensitivity, specificity, PPV, and NPV for identifying ALD were 1.00, 0.96, 0.19, and 1.00, respectively. With a cutoff value of 5 points for AUDIT-C, the sensitivity, specificity, PPV, and NPV for identifying ALD were 1.00, 0.75, 0.04, and 1.00, respectively. Similar results were observed for the ability of AUDIT and AUDIT-C to identify excessive alcohol intake (Supplementary Tables S4 and S5).

## Discussion

Regular health checkups at schools are an important opportunity to not only improve student welfare but also detect rising health disorders^16^. In Japan, regular health checkups for students based on the School Health and Safety Act are provided for students from preschool to university^16^. With the established health checkup system, the screening of MAFLD and ALD may contribute to early treatment intervention; thus, improving the outcomes of CLD. However, since MAFLD is a relatively newly defined term, very few attempts have been made to screen for MAFLD during health checkups, especially among young adults. Furthermore, the prevalence and screening methods for ALD in young adults remain unclear. Therefore, this study fills the gap between the rising etiologies of CLD and the use of regular health checkup in young adults for early detection and treatment/intervention to improve CLD outcomes.

Our study found a relatively high prevalence of NAFLD (17%), MAFLD (11%), ALD (1%), and excessive alcohol intake (4%) among the male graduate students. Japanese male young adults with NAFLD or MAFLD were mainly characterized by overweight/obesity and metabolic risk abnormalities, whereas none of them had type 2 diabetes. Furthermore, we demonstrated that serum ALT levels, BMI, and AUDIT are powerful screening parameters to identify high-risk male graduate students. This study, therefore, expanded our knowledge on the risks of CLD in Japanese male young adults and provided simple screening methods to identify students at risk for advanced CLD and severe outcomes.

As for the prevalence of NAFLD, a recent Japanese meta-analysis showed that the overall prevalence of NAFLD in Japan is 26% and males have a higher prevalence than females (34% vs. 16%)^17^. Furthermore, the prevalence of NAFLD in Japan is predicted to increase^17^. Despite the accumulated evidence of NAFLD prevalence, very few attempts have been made to determine at-risk individuals among young adults. The prevalence of NAFLD in our study (17%) seems reasonable, given that the Global Burden of Disease data have shown that the prevalence of NAFLD is approximately 9%, 16%, and 21% among ages 15–19, 20–24, and 25–29 years, respectively^4^. When comparing NAFLD with MAFLD in our study, the prevalence of MAFLD was lower than that of NAFLD among relatively young particpants^18^. However, MAFLD prevalence was higher than that of NAFLD in other studies with older participants^7,19^. Reviewing the results of our study with young participants and other studies with older participants, we assumed that metabolic changes due to aging are a strong driver of MAFLD and that the prevalence of MAFLD exceeds that of NAFLD around middle age. A large cohort study revealed that MAFLD, but not NAFLD, was associated with an increased risk of all-cause mortality^8^. Given the impact of MAFLD on outcomes, early detection and intervention of MAFLD, especially in young population, may play a critical role in preventing the future burden of CLD.

As MAFLD has a stronger impact on mortality than NAFLD^8^, effective and convenient markers for MAFLD are urgently required. For large-scale screening of MAFLD, serum biomarkers are reasonable and preferred because imaging techniques are impractical in terms of cost effectiveness and availability^11^. The guidelines of NAFLD recommend the FIB-4 index or NAFLD fibrosis score, both of which comprise serum ALT levels, to stratify the risk of advanced fibrosis and avoid unnecessary liver biopsy^10,11^. Although these scoring methods are useful and well-validated for screening patients with advanced fibrosis, their use in health checkup setting is not yet common because platelet count and serum albumin levels are not mandatory for school health checkup including university. Therefore, there is a need for a simple screening method to detect NAFLD and MAFLD in early stage to offer an opportunity to prevent disease progression.

Our study revealed that a simple measurement of serum ALT levels may be sufficient to detect high-risk populations and is an ideal biomarker to identify NAFLD and MAFLD in terms of AUCs. We also found that the optimal cutoff value of ALT showed relatively high sensitivity and specificity for identifying MAFLD in this study. There are only a few reports on noninvasive biomarkers of MAFLD, which are poorly investigated compared to that of NAFLD. Although liver function tests have been the most traditional approach to investigate CLD, recent guidelines have concluded that this strategy lacks evidence for both screening and diagnostic methods and requires further investigation^20^. However, our study provides meaningful evidence that measurement of serum ALT levels is an easy and practical approach for screening MAFLD among male young adults. The results of our study also strengthen the importance of biochemical analysis to stratify the risk of CLD during health checkups, even among young adults.

Another interesting finding in our study was that serum ALT levels were significantly higher in participants with overlapping conditions than in those with NAFLD only. Hepatocellular damage is the pathophysiological component of CLD and, therefore, serum ALT levels have a strong impact on liver-related and all-cause mortality^21^. Since obesity plays an important role in the development of nonalcoholic steatohepatitis^22^, our results may reflect that the pathogenesis of MAFLD has a stronger impact on hepatocellular damage than that of NALFD. This can be a reasonable explanation as previous studies have shown that MAFLD has a worse survival rate than NAFLD^8^. Therefore, it is reasonable to focus on MAFLD as a target for screening and intervention in the health checkup setting.

In addition to serum ALT levels, BMI was also a robust marker to identify MAFLD and NAFLD among Japanese male young adults in terms of AUCs. Interestingly, the cutoff values of BMI to identify MAFLD (22.9 kg/m^2^) and NAFLD (21.5 kg/m^2^) were lower than the cutoff value for obesity proposed by the Japan Society for the Study of Obesity (≥ 25 kg/m^2^)^23^. A recent study has suggested that even normal weight population can develop NAFLD because of fat accumulation and reduced muscle mass^24^. Since BMI strongly correlates with visceral adipose tissue^23^, our study suggests even nonobese male young adults can develop MAFLD and NAFLD, and BMI could be a robust marker to stratify the risk of these CLDs.

The prevalence of and screening methods for alcohol misuse and ALD among young male adults were investigated in our study. In general, the prevalence of excessive alcohol intake depends on the definition and cohort, and ranges from 10 to 40% in the Japanese population^14,25,26^. In addition, the prevalence of ALD in the Japanese population is around 2% based on a recent review^27^. In our study, approximately 4% of students consumed ≥ 20 g/day of alcohol, and 1% had ALD. A previous study of alcohol use in Japanese college students showed that 13% of students had excessive alcohol intake^28^. This discrepancy can be explained by the difference in region, academic degree, and decreased social opportunity for drinking because of coronavirus disease 2019. For screening, the AUDIT and AUDIT-C were the only methods used to identify the risk factors for cirrhosis. Ideally, all participants should be assessed by AUDIT for ALD screening; however, our data show that the AUDIT-C, a shortened AUDIT, can be a reasonable alternative considering the limited time available to complete health checkups. Accumulated evidence has shown that a high proportion of patients with ALD do not exhibit any clinical or biochemical abnormalities^13^, and our study also confirmed that there is no difference in biochemical parameters between participants with and without ALD in Japanese male young adults. Therefore, identifying excessive alcohol intake is a fundamental step for screening ALD. A recent Japanese nationwide survey revealed that cases of liver cirrhosis associated with ALD is increasing and, therefore, it is essential to identify alcohol misuse and ALD among young adults to reduce the burden of CLD^29^. This report contains the first evidence of the usefulness of AUDIT and AUDIT-C for screening ALD among Japanese young adults.

The clinical relevance of our findings should be explained. We found that health checkup is an important opportunity to identify, MAFLD, NAFLD, and ALD in Japanese male young adults. However, it is impractical to provide liver ultrasonography to all individuals based on our cutoff values because the values were relatively low considering the commonly used cutoff values^20,21^. In addition, ultrasonography is costly and requires well-trained examiner. Given that very few attempts have been made on the screening of CLD in younger population, we believe that our findings have meaningful implication as using health checkups to assess and inform individuals about their risk for CLD may help them to make lifestyle modification that reduces the future burden of CLD.

This study had several limitations. First, this was a single-center study that included only males and our results may not be applicable to other regions and females. Especially, females were initially excluded from the study considering the sex differences of obesity, type 2 diabetes, other metabolic abnormalities, and normative values of liver enzymes^20,21,30^. Second, liver biopsy was not performed in this study and liver steatosis was diagnosed only by ultrasonography. The small number of ALD cases may have limited the statistical power of our study. Third, we lacked data on well-validated scores, such as FIB-4 index and NAFLD fibrosis score, and future studies should evaluate the clinical relevance of these scores in younger populations. Therefore, a multicenter study that includes both males and females is required to validate our results. Despite these limitations, this study may have important implications for screening and identifying the prevalence of MAFLD and excessive alcohol intake among male young adults in a health checkup setting.

In conclusion, our study provides the first evidence of the prevalence of MAFLD and ALD in young Japanese male adults. Furthermore, simple measurements of serum ALT levels, BMI, AUDIC, and AUDIT-C are useful for screening CLDs. Our study may guide the establishment of strategies for MAFLD and ALD identification in younger generations, and confirm the importance of health checkups for early detection and intervention in reducing the future burden of CLD.

## Supplementary Information

Supplementary Information.

## Data Availability

The data analyzed in this study is availabule from the corresponding author on reasonable request.
