# Supplementary material for: Usefulness of health checkup for screening metabolic dysfunction-associated fatty liver disease and alcohol-related liver disease in Japanese male young adults
Source: Sci Rep. 2023 May 18;13:7987. doi: 10.1038/s41598-023-34942-x (PMC10195773; doi:10.1038/s41598-023-34942-x)
Supplement: Supplementary file 1 — Supplementary Information. [file 41598_2023_34942_MOESM1_ESM.pdf]

Supplementary Information

Title: Usefulness of health checkup for screening metabolic dysfunction-associated fatty liver disease and alcohol-related liver disease in Japanese male young adults

Satoko Tajirika[1, 2], Takao Miwa[1, 2*], Cathelencia Francisque[3], Tatsunori Hanai[2], Nanako Imamura[1], Miho Adachi[1], Ryo Horita[1], Lynette J Menezes[3], Masahito Shimizu[2], Mayumi Yamamoto[1, 4, 5]

[1] Health Administration Center, Gifu University, Gifu, Japan

[2] Department of Gastroenterology/Internal Medicine, Graduate School of Medicine, Gifu University, Gifu, Japan

[3] Morsani College of Medicine, University of South Florida, Tampa, Florida, United States of America

[4] Department of Diabetes and Metabolism, Gifu University Hospital, Gifu, Japan

[5] United Graduate School of Drug Discovery and Medical Information Sciences, Gifu University, Gifu, Japan

Supplementary Table S1. Characteristics of participants according to alcohol intake

Characteristic	Alcohol intake ≥ 20 g/day (n = 11)	Alcohol intake < 20 g/day (n = 302)	*P*-value*
**Demographics**			
Age (years)	24 (± 6)	23 (± 4)	0.169
Current/former smoking, n (%)	2 (18)	22 (7)	0.182
Exercise habits, n (%)	5 (45)	118 (39)	0.670
**Physical examination**			
Waist circumference (cm)	79 (± 7)	78 (± 8)	0.438
Body mass index (kg/m^2)	21.4 (± 2.7)	21.2 (± 2.9)	0.868
**Comorbidity**			
Diabetes, n (%)	0 (0)	0 (0)	NA
Overweight or obesity, n (%)	3 (27)	64 (21)	0.629
**Metabolic risk abnormality**			
High waist circumference, n (%)	2 (18)	26 (9)	0.275
High blood pressure, n (%)	3 (27)	73 (24)	0.814
High TG, n (%)	2 (18)	43 (14)	0.714
Low HDL-C, n (%)	0 (0)	13 (4)	0.482
Prediabetes, n (%)	0 (0)	6 (2)	0.637
**Laboratory test**			
AST (U/L)	21 (± 4)	21 (± 13)	0.084
ALT (U/L)	24 (± 9)	26 (± 22)	0.415
TG (mg/dL)	106 (± 45)	99 (± 71)	0.327
HDL-C (mg/dL)	62 (± 11)	58 (± 13)	0.156
LDL-C (mg/dL)	91 (± 23)	97 (± 26)	0.374
HbA1c (%)	5.2 (± 0.2)	5.2 (± 0.2)	0.772
MAFLD, n (%)	1 (9)	33 (11)	0.848
NAFLD, n (%)	1 (9)	52 (17)	0.480
**AUDIT**	16.0 (± 6.8)	3.4 (± 3.5)	< 0.001
Q1-Q3 (AUDIT-C)	9.0 (± 2.0)	2.7 (± 2.4)	< 0.001
Q4-Q6	2.5 (± 2.4)	0.3 (± 0.8)	< 0.001
Q7-Q10	4.4 (± 3.7)	0.4 (± 1.1)	< 0.001

Values are presented as numbers (percentages) or means (standard deviations).

*Statistical differences between the two groups were analyzed using the chi-square test or Mann-Whitney *U* test.

Abbreviations: ALT, alanine aminotransferase; AST, aspartate aminotransferase; AUDIT, alcohol use disorder identification test; AUDIT-C, alcohol use disorder identification test-consumption; HbA1c, hemoglobin A1c; HDL-C, high-density lipoprotein cholesterol; LDL-C, low-density lipoprotein cholesterol; MAFLD, metabolic dysfunction-associated fatty liver disease; NA, not available; NAFLD, non-alcoholic fatty liver disease; TG, triglycerides

Supplementary Table S2. Discriminative ability of each variable to identify MAFLD and NAFLD in participants

Characteristic	AUC (95% CI)	OR (95%CI)	*P*-value*
**MAFLD**			
Age	0.61 (0.51–0.70)	1.10 (1.03–1.16)	0.003
Current/former smoking	0.53 (0.49–0.56)	0.34 (0.04–2.58)	0.295
Excessive alcohol intake	0.50 (0.47–0.53)	0.82 (0.10–6.57)	0.848
Exercise habits	0.51 (0.42–0.59)	0.95 (0.46–1.98)	0.893
Waist circumference (cm)	0.87 (0.80–0.94)	1.19 (1.13–1.26)	< 0.001
Body mass index (kg/m^2)	0.94 (0.91–0.97)	2.08 (1.68–2.58)	< 0.001
AST (U/L)	0.76 (0.68–0.84)	1.03 (1.01–1.06)	0.004
ALT (U/L)	0.86 (0.80–0.92)	1.07 (1.04–1.09)	< 0.001
TG (mg/dL)	0.77 (0.67–0.86)	1.02 (1.01–1.02)	< 0.001
HDL-C (mg/dL)	0.71 (0.62–0.80)	0.92 (0.89–0.96)	< 0.001
LDL-C (mg/dL)	0.60 (0.50–0.71)	1.01 (1.00–1.02)	0.145
HbA1c (%)	0.71 (0.62–0.80)	38.93 (5.88–257.6)	< 0.001
AUDIT	0.50 (0.39–0.60)	1.00 (0.92–1.09)	0.982
AUDIT-C	0.50 (0.40–0.61)	1.00 (0.87–1.15)	0.978
**NAFLD**			
Age	0.55 (0.48–0.62)	1.07 (1.01–1.14)	0.018
Current/former smoking	0.50 (0.46–0.54)	0.98 (0.32–2.99)	0.971
Excessive alcohol intake	0.51 (0.49–0.53)	0.48 (0.06–3.84)	0.490
Exercise habits	0.51 (0.44–0.58)	0.92 (0.50–1.70)	0.799
Waist circumference (cm)	0.75 (0.67–0.83)	1.12 (1.08–1.16)	< 0.001
Body mass index (kg/m^2)	0.81 (0.74–0.88)	1.53 (1.34–1.74)	< 0.001
AST (U/L)	0.69 (0.61–0.77)	1.03 (1.01–1.05)	0.010
ALT (U/L)	0.76 (0.68–0.84)	1.06 (1.04–1.08)	< 0.001
TG (mg/dL)	0.69 (0.60–0.79)	1.01 (1.01–1.02)	< 0.001
HDL-C (mg/dL)	0.61 (0.53–0.70)	0.96 (0.94–0.99)	0.008
LDL-C (mg/dL)	0.51 (0.42–0.60)	1.00 (0.99–1.01)	0.770
HbA1c (%)	0.71 (0.64–0.78)	45.56 (8.54–242.97)	< 0.001
AUDIT	0.51 (0.43–0.59)	0.97 (0.90–1.05)	0.479
AUDIT-C	0.49 (0.41–0.58)	0.98 (0.87–1.09)	0.675

*Statistical analyses were performed using a logistic regression model and receiver operating characteristic curve.

Abbreviations: ALT, alanine aminotransferase; AST, aspartate aminotransferase; AUC, area under the curve; AUDIT, alcohol use disorders identification test; AUDIT-C, alcohol use disorder identification test-consumption; CI, confidence interval; HbA1c, hemoglobin A1c; HDL-C, high-density lipoprotein cholesterol; LDL-C, low-density lipoprotein cholesterol; MAFLD, metabolic dysfunction-associated fatty liver disease; NAFLD, non-alcoholic fatty liver disease; OR, odds ratio; TG, triglycerides

Supplementary Table S3. Discriminative ability of each variable in identifying ALD in participants

**Characteristic**	**AUC (95% CI)**	**OR (95%CI)**	***P*-value***
Age	0.49 (0.19–0.79)	0.87 (0.34–2.23)	0.768
Current/former smoking	0.54 (0.52–0.55)	NA	0.995
Exercise habits	0.70 (0.67–0.73)	NA	0.995
Excessive alcohol intake	0.99 (0.98–1.00)	NA	0.996
Waist circumference (cm)	0.43 (0.08–0.79)	1.03 (0.92–1.15)	0.622
Body mass index (kg/m^2)	0.55 (0.12–0.97)	1.08 (0.77–1.51)	0.669
AST (U/L)	0.62 (0.37–0.88)	1.00 (0.91–1.10)	0.960
ALT (U/L)	0.71 (0.51–0.91)	1.01 (0.97–1.05)	0.778
TG (mg/dL)	0.61 (0.24–0.99)	1.00 (0.99–1.01)	0.784
HDL-C (mg/dL)	0.63 (0.08–1.00)	1.02 (0.95–1.10)	0.554
LDL-C (mg/dL)	0.55 (0.05–1.00)	1.00 (0.95–1.05)	0.861
HbA1c (%)	0.61 (0.29–0.93)	0.17 (0.00–65.30)	0.559
AUDIT	0.98 (0.96–1.00)	1.29 (1.11–1.50)	0.001
AUDIT-C	0.92 (0.80–1.00)	1.85 (1.20–2.86)	0.005

*Statistical analyses were performed using a logistic regression model and receiver operating characteristic curve.

Abbreviations: ALD, alcohol-related liver disease; ALT, alanine aminotransferase; AST, aspartate aminotransferase; AUC, area under the curve; AUDIT, alcohol use disorders identification test; AUDIT-C, alcohol use disorder identification test-consumption; CI, confidence interval; HbA1c, hemoglobin A1c; HDL-C, high-density lipoprotein cholesterol; LDL-C, low-density lipoprotein cholesterol; NA, not available; OR, odds ratio; TG, triglycerides

Supplementary Table S4. Discriminative ability of each variable to identify excessive alcohol intake in participants

**Characteristic**	**AUC (95% CI)**	**OR (95%CI)**	***P*-value***
Age	0.60 (0.43–0.76)	1.05 (0.94–1.16)	0.393
Current/former smoking	0.55 (0.43–0.68)	2.83 (0.58–13.90)	0.201
Exercise habits	0.53 (0.38–0.69)	1.30 (0.39–4.35)	0.671
Waist circumference (cm)	0.57 (0.42–0.72)	1.02 (0.95–1.08)	0.636
Body mass index (kg/m^2)	0.52 (0.33–0.70)	1.02 (0.83–1.24)	0.863
AST (U/L)	0.65 (0.50–0.80)	1.00 (0.96–1.04)	0.864
ALT (U/L)	0.57 (0.43–0.72)	1.00 (0.96–1.03)	0.790
TG (mg/dL)	0.59 (0.41–0.76)	1.00 (0.99–1.01)	0.760
HDL-C (mg/dL)	0.63 (0.46–0.80)	1.02 (0.98–1.06)	0.313
LDL-C (mg/dL)	0.58 (0.39–0.77)	0.99 (0.96–1.01)	0.355
HbA1c (%)	0.53 (0.34–0.71)	0.79 (0.04–16.82)	0.881
AUDIT	0.97 (0.94–1.00)	1.49 (1.28–1.74)	< 0.001
AUDIT-C	0.97 (0.94–1.00)	2.81 (1.80–4.39)	< 0.001

*Statistical analyses were performed using a logistic regression model and receiver operating characteristic curve.

Abbreviations: ALT, alanine aminotransferase; AST, aspartate aminotransferase; AUC, area under the curve; AUDIT, alcohol use disorders identification test; AUDIT-C, alcohol use disorder identification test-consumption; CI, confidence interval; HbA1c, hemoglobin A1c; HDL-C, high-density lipoprotein cholesterol; LDL-C, low-density lipoprotein cholesterol; OR, odds ratio; TG, triglycerides

Supplementary Table S5. Ability of AUDIT and AUDIT-C to identify excessive alcohol intake in participants

**Screening item**	**OR (95% CI)**	***P*-value***	**AUC (95% CI)**	**Optimal cutoff**	**Sensitivity**	**Specificity**	**PPV**	**NPV**
AUDIT	1.49 (1.28–1.74)	< 0.001	0.97 (0.94–1.00)	7	1.00	0.84	0.18	1.00
AUDIT-C	2.81 (1.80–4.39)	< 0.001	0.97 (0.94–1.00)	7	0.91	0.93	0.31	1.00

*Statistical analyses were performed using a logistic regression model and receiver operating characteristic curve.

Abbreviations: AUC, area under the curve; AUDIT, alcohol use disorders identification test; AUDIT-C, alcohol use disorders identification test-consumption; CI, confidence interval; NPV, negative predictive value; OR, odds ratio; PPV, positive predictive value
